# Plasma serpinB1 is related to insulin sensitivity but not pancreatic *β*‐Cell function in non‐diabetic adults

**DOI:** 10.14814/phy2.13193

**Published:** 2017-03-14

**Authors:** Michael Glicksman, Asha Asthana, Brent S. Abel, Mary F. Walter, Monica C. Skarulis, Ranganath Muniyappa

**Affiliations:** ^1^ Diabetes, Endocrinology and Obesity BranchNIDDKNIHBethesdaMaryland; ^2^ Clinical Core LaboratoryNational Institute of Diabetes and Digestive and Kidney DiseasesNational Institutes of HealthBethesdaMaryland

**Keywords:** Insulin resistance, Insulin sensitivity, SerpinB1, *β*‐cell function, *β*‐cell proliferation

## Abstract

Pancreatic *β*‐cell dysfunction because of reduced *β*‐cell mass and function is a primary determinant in the progression of diabetes. Increase in *β*‐cell mass and compensatory hyperinsulinaemia is frequently associated with insulin‐resistant states. Although the humoral factors mediating this compensatory response are unknown, serpinB1, a protease inhibitor, has recently been proposed to be one such factor. In this study, we examine the relationships between plasma serpinB1, insulin sensitivity, and pancreatic *β*‐cell function in non‐diabetic individuals. 117 subjects (women, *n* = 50, men, *n* = 67; age= 37.6 ± 10.8; BMI=31.1 ± 7.7 kg/m^2^) underwent an insulin‐modified frequently sampled intravenous glucose tolerance test (FSIVGTT) at the NIH Clinical Research Center. Acute insulin response (AIR) and insulin sensitivity index (SI) were obtained from the FSIVGTT with MINMOD analysis. The Quantitative Insulin Sensitivity Check Index (QUICKI) was calculated from fasting insulin and glucose values. Plasma serpinB1 levels were measured using an ELISA assay. Simple linear correlation analyses were performed to evaluate the relationship between serpinB1 and measures of insulin sensitivity and *β*‐cell function. Circulating serpinB1 levels were unrelated to age, sex, race, BMI, or percent body fat. SI but not AIR significantly correlated with circulating serpinB1 levels (*r* = 0.23, *P* < 0.05). QUICKI tended to positively correlate with serpinB1 (*r* = 0.16, *P* = 0.09). Circulating serpinB1 is directly associated with insulin sensitivity but not *β*‐cell function in non‐diabetic adults. Whether this modest association plays a role in insulin sensitivity in humans remains to be clarified.

## Introduction

In insulin‐resistant states, compensatory changes in *β*‐cell mass and function are critical in maintaining normal plasma glucose homeostasis (Weir et al. 2001). Previous reports have focused on identifying potential endocrine/autocrine factors responsible for the compensatory *β*‐cell proliferation in response to diminished insulin sensitivity (Sachdeva and Stoffers 2009). Metabolites and peptides secreted by other organs, hormones derived from adipose tissue, as well as signals secreted by muscle and bone, have all been proposed as potential circulatory factors responsible for this phenomenon (Sachdeva and Stoffers 2009; El Ouaamari et al. 2013; Dirice et al. 2014). Liver‐specific insulin receptor knockout in mice (LIRKO) upregulates pancreatic *β*‐cell proliferation (El Ouaamari et al. 2013; Dirice et al. 2014). Using this model, El Ouaamari et al. (2016), recently demonstrated serpinB1 as the hepatocyte‐derived factor responsible for this *β*‐cell proliferation.

Leukocyte elastase inhibitor, more commonly referred to as serpinB1, is an endogenous protease inhibitor (Cooley et al. 2001). This liver‐derived secretory protein belongs to the serpin superfamily of serine proteinase inhibitors. Previous researchers have studied the role of serpinB1 in immunologic responses and as a diagnostic biomarker (Silverman et al. 2001; Tseng et al. 2009; Benarafa et al. 2011). Whether circulating serpinB1, a newly identified “betatrophin” is related to *β*‐cell function in humans is unknown. To that end, in this cross‐sectional study, we sought to explore the relationship between serpinB1, insulin sensitivity, and *β*‐cell function in a lean/overweight/obese but otherwise healthy adult population.

## Subjects and Methods

### Study design and study subjects

The Institutional Review Board of the National Institutes of Diabetes and Digestive and Kidney Diseases (NIDDK) approved the study protocol (ClinicalTrials.gov identifier NCT00428987). In this report, we examined the association between serpinB1, insulin sensitivity, and *β*‐cell function in non‐diabetic subjects as an exploratory outcome. Consequently, the study population was a subset from an ongoing study titled, “Physical and Behavioral Traits of Overweight and Obese Adults” (NCT00428987). The subset of subjects from the original cohort who were non‐diabetic and not on any medications that affected insulin sensitivity or glucose metabolism were included in this study. Written informed consent was obtained from all participants. Patients over the age of 18 years and a BMI ≥ 18.5 kg/m^2^ were eligible to participate. All subjects were admitted to the Metabolic Clinical Research Unit at the NIH Clinical Center for one or more days. During the admission, routine fasting plasma samples were collected and stored at −70°C by the NIDDK Clinical Core. All clinical chemistry testing was performed by the Department of Laboratory Medicine, NIH Clinical Center.

### SerpinB1 measurements

SerpinB1 was measured in plasma using a commercially available ELISA using an antibody that recognizes AA 167‐196 in the middle region of human serpinB1 (antibodies‐online Inc., Atlanta, GA). Plasma samples were not diluted ahead of the ELISA and each sample was measured in duplicate. Specificity of the ELISA was examined by substituting samples with 10 ng/mL of recombinant human serpinB1 with an N‐terminal polyhistidine tag (OriGene, Rockville, MD).

### Metabolic testing

Each participant underwent a frequently sampled intravenous glucose tolerance test (FSIVGTT) during their inpatient admission as previously described (Bergman et al. 1979; Muniyappa et al. 2012). After a 12 h overnight fast, an intravenous bolus of glucose (0.3 g/kg) was administered over one to two minutes. In addition, a bolus of intravenous insulin (0.03 U/kg) was administered at time 20 min. Blood samples for glucose and insulin measurements were taken at −10, −1, 0, 1, 2, 3, 4, 5, 6, 7, 8, 10, 12, 14, 16, 20, 22, 23, 24, 25, 27, 30, 40, 50, 60, 70, 80, 90, 100, 120, 150, and 180 min. Insulin sensitivity index (SI) and acute insulin response (AIR) were calculated using MinMod Millenium program (Bergman et al. 1979; Muniyappa et al. 2012). AIR, a surrogate marker of *β*‐cell function, was calculated as the area under the curve for plasma insulin concentrations between 0 and 10 min for the insulin concentration above basal (average of −10, −1, and 0 min time points). However, to account for the prevailing level of insulin resistance, disposition index (DI) was calculated (product of AIR and SI). Quantitative Insulin Sensitivity Check Index (QUICKI), a surrogate marker for insulin sensitivity, was calculated as defined previously from fasting glucose and insulin values (Muniyappa et al. 2008). QUICKI = 1/(log[I0] + log[G0]), where I0 is fasting insulin (*μ*U/mL) and G0 is fasting glucose (mg/dL). Percent body fat was measured using dual energy x‐ray absorptiometry (Lunar iDXA, GE Healthcare, Madison, WI).

### Statistical analysis

Descriptive analysis results are reported as mean and standard deviation. Normality was checked using D'Agostino and Pearson omnibus normality test and skewed variables were natural logarithm transformed as needed. Differences in serpinB1 levels between sexes and racial groups were evaluated using unpaired t‐test. Bivariate regressions and Pearson's correlation coefficient were used to assess the associations between serpinB1 and various metabolic parameters. All statistical analyses were performed using Graph Pad Prism 6.

## Results

### Baseline clinical characteristics of study subjects

In a cross‐sectional study design, 117 subjects (women, *n* = 50; men, *n* = 67) of mean 38 years of age were studied. Clinical characteristics of the cohort are reported in Table 1. All participants were healthy, weight‐stable, and non‐diabetic. Metabolic parameters including lipid profiles are reported in Table 1. Participants were non‐diabetic with normal fasting glucose and lipid parameters. Median values of QUICKI and SI suggests a moderately insulin‐sensitive group but with a wide spectrum (SI: 0.2–17.9 [(*μ*u/l)^−1^ min^−1^). Similarly, mean AIR (686 *μ*U·mL^−1^·min^−1^) during an FSIVGTT was robust with a range of 17–3850 *μ*U·ml^−1^·min^−1^. As expected, SI was negatively associated with BMI (*r* = −0.58, *P* < 0.001), total body fat percent (*r* = −0.42, *P* < 0.001) and AIR (*r* = −0.54, *P* < 0.001). Likewise, AIR, a measure of *β*‐cell function was related to BMI (*r* = 0.28, *P* < 0.01), total body fat percent (*r* = 0.20, *P* < 0.05), and A1C (*r* = −0.21, *P* < 0.05).

**Table 1:** Clinical and metabolic characteristics of the study participants

Clinical Parameters	
Age (years)	36 (29–45)
Sex	67 M, 50 F
Race (W: Caucasian, AA: African American)	53 W, 64 AA
BMI (kg/m^2^)	31.1 ± 7.8
Body fat (%)	33.7 ± 11.4
SerpinB1 (ng/mL)	2.14 ± 3.95
AIR (*μ*U·mL^−1^·min^−1^)	686 ± 681
SI [(mu/L)^−1 ^min^−1^]	4.1 ± 3.2
DI (SI × AIR)	1857 ± 1426
Total Cholesterol (mg/dL)	173 ± 32
HDL (mg/dL)	53 ± 14
LDL (mg/dL)	99 ± 29
Triglycerides (mg/dL)	100 ± 58
Fasting glucose (mg/dL)	89 ± 10
Fasting insulin (*μ*U/mL)	9.8 ± 10.3
Hemoglobin A1C (%)	5.5 ± 0.4
QUICKI	0.361 ± 0.044

Values shown are mean ± SD. AIR, acute insulin response; DI, disposition index; SI, insulin sensitivity index; and QUICKI, quantitative insulin sensitivity check index.

### SerpinB1 and metabolic parameter associations

**Figure 1:**
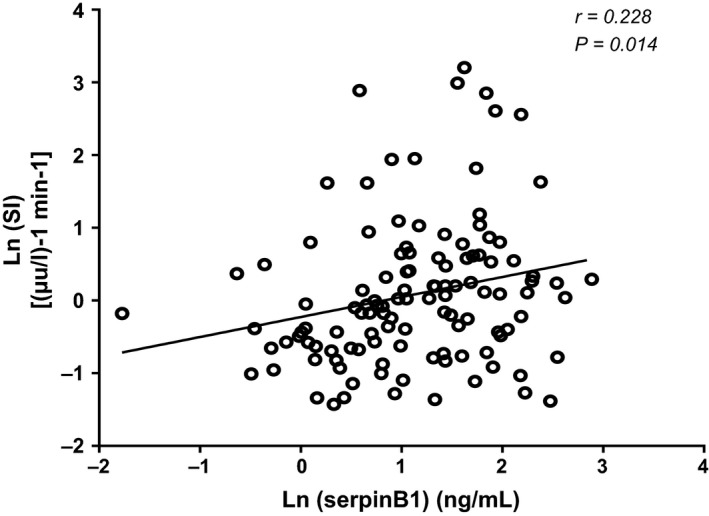
Linear correlations between plasma serpinB1 and insulin sensitivity index (SI) in 117 non‐diabetic adult individuals. Both serpinB1 levels and SI are natural log transformed. Pearson correlation coefficient (r) and corresponding *P* values are shown.

Circulating serpinB1 levels as measured by an ELISA were 2.14 ng/mL ± 3.95 ng/mL (mean ± SD). Bivariate regressions between serpinB1 and various parameters are reported in Table 2. Circulating serpinB1 levels were unrelated to age, sex, race, BMI, or percent body fat. SI was significantly correlated with circulating serpinB1 levels (*r* = 0.23, *P* < 0.05). However, QUICKI tended to positively correlate with serpinB1(*r* = 0.16, *P* = 0.09) (Fig. 1). AIR was unrelated to serpinB1 levels (Table 2). No other metabolic parameters were associated with serpinB1 levels.

**Table 2:** Relationships between SerpinB1 and various parameters

	R
Age	−0.04
BMI (kg/m^2^)	−0.04
% Body fat	0.05
AIR	−0.13
**SI**	**0.23** *
DI	0.06
Fasting glucose	−0.06
Fasting insulin	−0.15
QUICKI	0.16

Pearson correlation coefficients between serpinB1 and various metabolic parameters. *P < 0.05 was considered to represent a statistically significant relationship. AIR, acute insulin response; SI, insulin sensitivity index; DI, disposition index; and QUICKI, quantitative insulin sensitivity check index.

## Discussion

In this study of healthy non‐diabetic participants, serpinB1 was positively associated with insulin sensitivity derived from the FSIVGTT and unrelated to *β*‐cell function. In studies by El Ouaamari et al. (2016), serpinB1 levels were higher in liver and serum of liver insulin receptor knockout mice (LIRKO) compared with wild type. In vitro, recombinant human serpinB1 stimulated *β*‐cell proliferation in mouse and human islets. SerpinB1 knockout in mouse models of insulin resistance attenuated compensatory *β*‐cell proliferation. Finally, small molecule serpinB1 mimetics enhanced *β*‐cell proliferation. Based on these findings, serpinB1 has been proposed as a hepatic factor that is upregulated in insulin‐resistant states, to stimulate compensatory pancreatic *β*‐cell proliferation (El Ouaamari et al. 2016).

Plasma serpinB1 levels were measured in individuals at risk for developing type 2 diabetes mellitus (*n* = 49) (El Ouaamari et al. 2016). The concentration of serpinB1 in this cohort varied with a range of 4–56 ng/mL. In a multivariate analysis, circulating serpinB1 levels were modestly but positively correlated with BMI and insulin resistance, consistent with the idea that insulin resistance accentuates serpinB1 levels. Insulin sensitivity in this study (El Ouaamari et al. 2016) was measured by composite insulin sensitivity index (Matsuda Index) (Matsuda and DeFronzo 1999). However, the authors did not measure *β*‐cell function or insulin secretion. Contrasting these findings, we report that serpinB1 levels positively correlate, albeit modestly with insulin sensitivity and are unrelated to AIR in non‐diabetic individuals. SI is a dynamic measure of insulin sensitivity in contrast to QUICKI which is a fasting surrogate of insulin sensitivity. The relationship between serpinB1 and QUICKI was not significant (*P* = 0.09); this could be because of the variability in the measurement of QUICKI, the surrogate nature of the index, or that QUICKI may represent hepatic insulin sensitivity. It is worth noting that in this cohort, both SI and AIR exhibit associations with BMI and total body fat that are well established. This suggests that the range of insulin sensitivity and measure of *β* ‐cell function was sufficient to test our hypothesis. Rodent studies suggest that serpinB1 augments pancreatic *β*‐cell proliferation and thus plasma levels may correlate with pancreatic *β*‐cell mass (El Ouaamari et al. 2016). However, it is worth noting that AIR is a measure of first‐phase insulin secretion and not a surrogate of *β*‐cell mass. Whether serpinB1 levels correlates with first‐ or second‐phase insulin secretion is not known. Unfortunately, we did not measure C‐peptide levels in this study and hence were unable to obtain more detailed indices of *β*‐cell function.

The reasons for the discrepancy between our study and the previous finding are unclear. However, differences in sample size, heterogeneity of the study population, methods to assess insulin sensitivity and serpinB1 assays may have contributed to the discrepancy. Our study cohort was larger, ethnically more diverse, and included more men. We measured insulin sensitivity using the widely used minimal model technique (FSIVGTT) in contrast to the highly variable Matsuda index (Muniyappa et al. 2008). The minimal model is excellent at predicting glucose disappearance during the FSIVGTT. Both indices of insulin sensitivity and *β*‐cell function can be simultaneously obtained in this dynamic test, rendering this an ideal and viable approach to test our hypothesis (Muniyappa et al. 2008). Feasibility of the more invasive and laborious hyperglycemic clamp technique, the gold standard technique to assess both insulin sensitivity and *β*‐cell function precluded us from using it in our study (Elahi 1996). Nonetheless, the wide range of BMI (range: 19–60 kg/m^2^), body fat (7–57%) and insulin sensitivity (SI: 0.2–17.9 [(*μ*u/L)^−1^ min^−1^]) of subjects in our cohort afforded us to better assess the relationships with serpinB1. In the prior study, serpinB1 ELISA assays were developed by the authors and had an intra‐ and inter‐assay variation of 13.6% and 16.4%, respectively. In our study, we used a commercial assay with an intra‐ and inter‐assay variation of 9.54% and 10.68%, respectively, and minimum detectable concentration of 0.24 ng/mL. Moreover, we independently confirmed the specificity of the commercial assay by spiking the plasma with recombinant human serpinB1. In a recent study, comparing serpinB1 levels in type 2 diabetic patients (*n* = 30) and healthy controls (*n* = 10), plasma serpinB1 levels measured using a commercial ELISA kit were (10.01 ± 3.59 [range with 1.93–17.09] vs. 5.69 ± 1.64 ng/mL [range with 2.79–8.40]), respectively (Takebayashi et al. 2016). Thus, serpinB1 levels in our study are consistent with values reported in this recent study.

In conclusion, we demonstrate that plasma serpinB1 levels are weakly associated with insulin sensitivity but not insulin secretion in non‐diabetic individuals. Our results do not support the theory that circulating serpinB1 is a marker of insulin resistance and thus may play a role in compensatory hyperinsulinemia in humans. Whether, plasma serpinB1 plays a role in insulin action or pancreatic *β*‐cell function in humans is unknown and remains to be determined.

## Conflict of Interest

There are no potential conflicts of interest relevant to this article.
